# Environmentally Friendly Surface Modification Treatment of Flax Fibers by Supercritical Carbon Dioxide

**DOI:** 10.3390/molecules25030438

**Published:** 2020-01-21

**Authors:** Maria Carolina Seghini, Fabienne Touchard, Laurence Chocinski-Arnault, Vincent Placet, Camille François, Laurent Plasseraud, Maria Paola Bracciale, Jacopo Tirillò, Fabrizio Sarasini

**Affiliations:** 1 Department of Chemical Engineering Materials Environment, Sapienza-Università di Roma, 00184 Roma, Italy; mariapaola.bracciale@uniroma1.it (M.P.B.); jacopo.tirillo@uniroma1.it (J.T.); 2 Département Physique et Mécanique des Matériaux, ENSMA, Institut PPRIME, CNRS-ENSMA-Université de Poitiers, 86961 Futuroscope CEDEX, France; fabienne.touchard@ensma.fr (F.T.); laurence.chocinski@ensma.fr (L.C.-A.); 3 Department of Applied Mechanics, CNRS/UFC/ENSMM/UTBM, FEMTO-ST Institute, University of Bourgogne Franche-Comté, 25030 Besançon, France; vincent.placet@univ-fcomte.fr (V.P.); camille.francois@u-bourgogne.fr (C.F.); 4 Catalysis, and Stereochemistry Team—OCS, CNRS/UB, Organometallics, ICMUB Institute, University of Bourgogne Franche-Comté, 21000 Dijon, France; Laurent.plasseraud@u-bourgogne.fr

**Keywords:** flax fibers, supercritical carbon dioxide, natural fibers, interface/interphase, surface modification treatments

## Abstract

The present work investigates the effects of an environmentally friendly treatment based on supercritical carbon dioxide (scCO_2_) on the interfacial adhesion of flax fibers with thermoset matrices. In particular, the influence of this green treatment on the mechanical (by single yarn tensile test), thermal (by TGA), and chemical (by FT-IR) properties of commercially available flax yarns was preliminary addressed. Results showed that scCO_2_ can significantly modify the biochemical composition of flax fibers, by selectively removing lignin and hemicellulose, without altering their thermal stability and, most importantly, their mechanical properties. Single yarn fragmentation test results highlighted an increased interfacial adhesion after scCO_2_ treatment, especially for the vinylester matrix, in terms of reduced debonding and critical fragment length values compared to the untreated yarns by 18.9% and 15.1%, respectively. The treatment was less effective for epoxy matrix, for which debonding and critical fragment length values were reduced to a lesser extent, by 3.4% and 3.7%, respectively.

## 1. Introduction

The development of natural fiber composites is currently under the spotlight not only in the academic but also in the industrial sector due to a host of reasons including their low density, low environmental impact, and competitive specific mechanical properties [1]. In this regard, flax fibers show a favorable combination of properties that make them a material of choice to reinforce polymers, such as their wide availability throughout the world, their morphology characterized by significant length (around 2–5 cm) with thick secondary walls (5–15 µm), good mechanical properties caused by a small microfibrillar angle, and high cellulose content and low density [2]. Therefore, flax fibers have been extensively investigated over the last twenty years [3,4,5], and two issues have been highlighted that still prevent their full exploitation as reinforcement in composite materials, i.e., the variability in properties and the poor interfacial adhesion with most polymer matrices. As regards the first issue, recent studies have demonstrated that tensile properties of flax fibers are not significantly affected over time irrespective of the fiber yield and variety and that the scattering in the specific mechanical properties lies in the same range of glass fibers [6]. According to Baley et al. [7], excellent composite properties with reduced variability can be reached if the fiber supply chain is carefully controlled and the manufacturing processes are optimized.

The quality of the fiber/matrix interface is another topic of considerable interest, as the poor compatibility with polymer matrices represents a major limitation for the industrial exploitation of natural fibers. Their peculiar hydrophilic surfaces prevent them from achieving a proper interfacial adhesion with the more hydrophobic polymer matrices, which leads to poor mechanical properties of the resulting composites. In addition, moisture retention can trigger dimensional changes, thus reducing the mechanical performance and increasing the proneness to microbiological degradation [8]. While numerous studies are still addressing the surface modification of natural fibers with a view to making them as much as possible compatible with composite materials requirements [9,10,11], their industrial implementation is compromised by the need to manage the large amounts of chemicals involved. These surface modification treatments range from chemical treatments [12,13,14,15,16,17] to physical treatments [18,19,20]. In particular, plasma modification of the surface of natural fibers can not only cause etching of the material and a related better physical adhesion but also the addition of free radicals. A further possibility is represented by the use of polymerizing gases, thus inducing a plasma polymerization or grafting on the fibers’ surface, as recently demonstrated for flax fibers with a tetravinylsilane coating deposited via Plasma Enhanced Chemical Vapour Deposition (PECVD) [21]. This treatment was able to increase the interfacial shear strength values by 114% and 71% in epoxy and vinylester composites, respectively. A more recent approach deals with the grafting of nanostructures on fibers’ surface to increase the adhesion with the polymer matrix [22,23,24] and/or induce multifunctionalities [25,26]. Ajith et al. [24] decorated the flax fiber yarns with hydrous zirconia nanoparticles by hydrolysis of zirconium oxychloride solution and by single fiber pull-out tests with an epoxy matrix reported an increase in the bonding strength by 65%. Wang et al. [27] grafted TiO_2_ nanoparticles on flax fibers and with the optimized amount of 2.34 wt% they obtained enhancements in both tensile strength of the flax fibers and interfacial shear strength with epoxy resin by 23.1% and 40.5%, respectively. In Reference [22], the authors developed a simple spray-coating process to deposit carbon nanotubes (CNTs) over the surface of ramie fibers. This coating was found to enhance the flexural strength and modulus of an epoxy-based composite by 38.4% and 36.8%, respectively, while a microdebonding test highlighted an increase in the interfacial shear strength by 25.7%. Sarker et al. [23] have recently reported the coating of graphene oxide (GO) and graphene flakes (G) on jute fibers and an interfacial shear strength enhancement by ∼236% compared to untreated fibers. However, these processes are often complex because there is a need to avoid nanostructures’ aggregation.

An envisaged challenge is to develop a pretreatment able to tailor the properties of natural fibers for their subsequent successful application in high-performance plant fiber composites but with limited costs and environmental impact. A less investigated approach deals with the use of supercritical fluids, which are substances above critical conditions (temperature and pressure) that show properties (e.g., density, viscosity, diffusion coefficient) intermediate between those of liquids and gases [28]. These fluids provide uncommon possibilities for selective extractions and fractionations, separation and purification, materials processing, and synthesis [29,30,31,32].

Among the different supercritical fluids, supercritical CO_2_ (scCO_2_) has attracted considerable interest because of its relatively moderate critical temperature of 31.1 °C and pressure of 73.8 bar [33]. scCO_2_ is a green attractive alternative to chemical solvents because it is inexpensive, essentially nontoxic, nonflammable, and can be easily recovered and recycled after use [28]. The interactions of supercritical fluids with lignocellulosic materials have been the subject of extensive investigations. The extent of dissolutions in wood species was investigated by Li and Kiran [33], and the authors reported that the extractions in carbon dioxide, ethylene, nitrous oxide, and n-butane were not significant. In Reference [34], the damage in several man-made and cellulose fabrics (cotton, viscose) after treatment in supercritical CO_2_ was assessed. The authors reported no shrinkage of the fabrics, but stress–strain measurements showed decreased tensile strength and elongation after treatment in carbon dioxide for 4 h at 160 °C and 300 bar. Depolymerization of cellulosic fibers was suggested as the result of a combination of heat, supercritical carbon dioxide, and treatment time due to cleavage of acetal bonds. This study highlights potential treatments of cellulose fibers (cotton and viscose) up to 140 °C or 160 °C but for a shorter time. Daza Serna et al. [35] used scCO_2_ for the pretreatment of lignocellulosic rice husk (80 °C, 270 bar, 10 min). The authors reported a significant delignification effect but no reductions in crystallinity and enzymatic digestibility.

Much limited attention has received the use of scCO_2_ for the surface treatment of plant fibers intended as reinforcement of polymer matrices. Gutiérrez et al. [36] studied the effect of supercritical carbon dioxide on the properties of Curauá fibers. The treatment with supercritical CO_2_ resulted in the partial lignin extraction from the fibers and fiber fibrillation. This result was confirmed by a FT-IR and thermogravimetric analysis. In particular, the untreated Curauá fibers showed the presence of three steps of mass loss: a first stage at a temperature of 100 °C, attributed to the presence of moisture in the fiber; a second stage around 300 °C associated with the hemicellulose and cellulose decomposition; and a final step of mass loss at a temperature of 400 °C, linked to the lignin decomposition and the production of volatile substances. The thermogravimetric curves found for the scCO_2_ treated fibers showed only the presence of the first two decomposition steps, confirming the lignin extraction produced by the supercritical solvent. Two series of biocomposites, one plasticized with dioctyl phthalate (DOP) and another with triethyl citrate (TEC), were prepared by extrusion. Fibrillation and uniform distribution of fibers in the cellulose acetate matrix were observed for both biocomposites.

Recently, a supercritical CO_2_ treatment has been developed in order to reduce the hygroscopic behavior and modify the physicochemical properties of hemp fibers [37,38]. The authors reported a decrease in the hygroscopic behavior of hemp fibers after the supercritical CO_2_ treatment ascribed to the extraction of polysaccharides from the fiber structure, such as hemicellulose and pectin, rich in hydroxyl groups. In addition, a decrease in the tensile stiffness of the fibers, as well as a more brittle fracture, was detected, coupled with a better fiber fineness.

These preliminary studies confirm that scCO_2_ treatment may represent a promising solution in order to tailor the adhesion of natural fibers with polymer matrices, even if the knowledge of the effects of such treatment on the mechanical, thermal, and morphological properties of natural fibers needs to be analyzed in detail. In this framework, the aim of the present work was to assess the suitability of a supercritical carbon dioxide treatment to improve the interfacial adhesion of flax yarns with two different thermoset matrices, namely epoxy and vinylester, with a view to designing chemically efficient products and processes that reduce or eliminate the use or generation of hazardous substances. The impact of this green treatment was evaluated by performing chemical, mechanical, thermal, and morphological characterizations at the yarn level and by micromechanical single-fiber fragmentation tests (SFFT) for interfacial quality assessment.

## 2. Results and Discussion

### 2.1. Characterization of Flax Yarns after scCO_2_ Treatment

**Figure 1:**
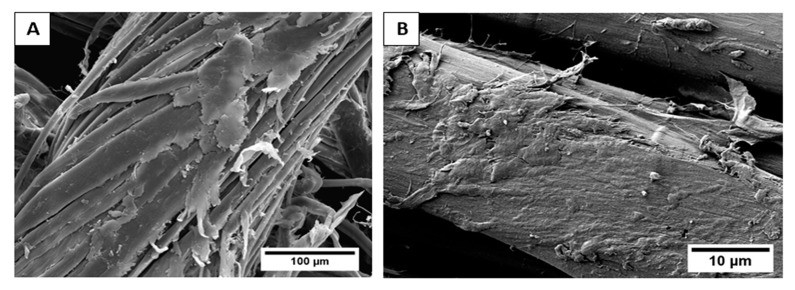
SEM micrographs showing the untreated flax yarn (**A**) and a detail of the flax fiber surface (**B**).

**Figure 2:**
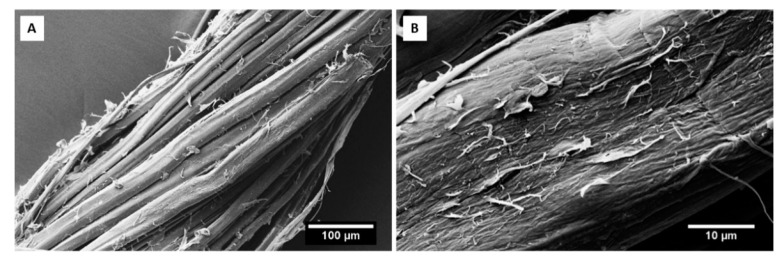
SEM micrographs showing the supercritical CO_2_ treated flax yarn (**A**) and a detail of the flax fiber surface (**B**).

The morphology of flax yarns after the supercritical carbon dioxide treatment was preliminarily investigated by scanning electron microscopy and the results are included in Figure 1 and Figure 2 for untreated and scCO_2_ treated flax yarns, respectively. Comparing the surface of the untreated flax yarn (Figure 1) and scCO_2_ treated flax yarn (Figure 2), it is possible to observe that the supercritical carbon dioxide treatment was able to remove impurities from the yarn surface. The wax-like substances decreased after the treatment, producing a relatively clean flax yarn surface, but at higher magnification, a phenomenon of surface peeling off was noted (Figure 2B). As reported by Zhang et al. [39], this peeling effect is due to changes to the surface morphology of the flax fibers occurring in supercritical carbon dioxide and that non-cellulosic constituents such as lignin, pectin, and hemicellulose have been removed in supercritical carbon dioxide fluid, as supported by the subsequent FT-IR results. In this regard, François et al. [38] reported a decrease in lignin content of hemp fibers up to 24.5% after scCO_2_ treatment. 

**Figure 3:**
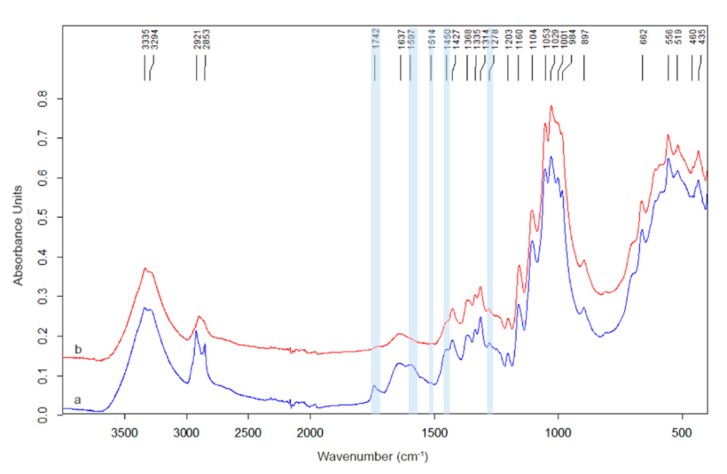
FT-IR spectra of as-received (a) and supercritical CO_2_ treated flax yarns (b). The modifications resulting from the treatment are highlighted in light blue.

The effect of the chemical treatment on the surface composition of flax fibers was studied by Fourier-Transform Infrared (FT-IR) analysis. A comparison between the FT-IR spectra of as-received and scCO_2_ treated flax yarns is shown in Figure 3.

The spectral analysis revealed that the untreated flax fiber consists of alkene, esters, aromatics, ketone, and alcohol belonging to cellulose, hemicellulose, and lignin (Table 1), as already reported in [21,40].

**Scheme 1:**
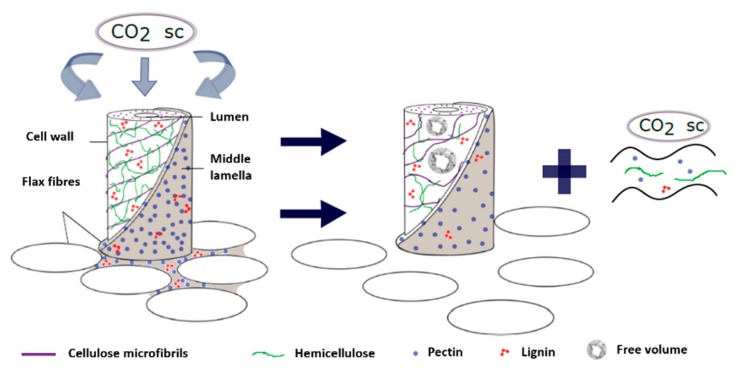
Effects of scCO_2_ treatment on flax fibers.

Upon CO_2_ supercritical treatment, slight shifts could be observed for the characteristic bands of flax. The stretching vibration of C–O–C and the bending vibration of C–H were shifted from 1161 cm^−1^ to 1157 cm^−1^ and from 897 cm^−1^ to 896 cm^−1^, respectively. Furthermore, a visible reduction of the bands at 1742 cm^−1^ and 1248 cm^−1^ of hemicellulose [41] and at 1597 cm^−1^, 1514 cm^−1^, and 1450 cm^−1^ of lignin was observed. These results confirm the removal of amorphous non-cellulosic compounds produced by supercritical CO_2_. Supercritical carbon dioxide fluid can infiltrate into flax fibers while causing their swelling, which favors the re-arrangements and re-crystallization of the molecule chains, thus inducing the shifts of the characteristic bands of treated flax fibers. On the other hand, the lignin structure in the flax fibers was easily destroyed and hydrolyzed in supercritical carbon dioxide. Carbon dioxide can also have a plasticization effect on flax fibers in the supercritical state and the increased mobility of the macromolecular chains can induce their rearrangement under high-temperature exposure, which should result in an increase in the crystallinity index [39,42]. These assumptions are confirmed by the values of the crystallinity index (CI) of cellulose calculated for the different flax yarns. The CI is an important parameter that refers to the relative crystallinity of the fiber samples, and the higher the value of CI, the greater the crystalline cellulose content and thus, the greater the efficiency of the scCO_2_ treatment in removing the amorphous components from flax [43]. Semiquantitative measurement of the crystallinity index of cellulose was evaluated as the intensity ratio between the IR absorption bands at 1427 and 897 cm^−1^ (I_1427_/I_897_), which are assigned to CH_2_ bending mode of cellulose and of the CH in β-glycosidic linkages between monosaccharides [44]. The cellulose IR crystallinity index calculated after the CO_2_ treatment increased by more than 65% (1.68) compared to untreated flax fiber (1.01), values that allowed a comparison between the untreated and treated states of flax yarns and confirmed the removal of amorphous constituents. The same conclusions were drawn in Reference [38], where the authors measured, by chemical analysis, a significant reduction after supercritical treatment in the content of three monosaccharides, namely rhamnose (Rha), galacturonic acid (GalA), and xylose (Xyl). Rha and GalA are the main components of pectin, while Xyl is linked to hemicellulose. NMR analysis performed on extracts collected in the supercritical carbon dioxide treatment procedure showed the presence of lignins and monosaccharide composition of isolated compounds [39]. A summary of the effects produced by the scCO_2_ treatment on flax fibers is schematically reported in Scheme 1.

**Figure 4:**
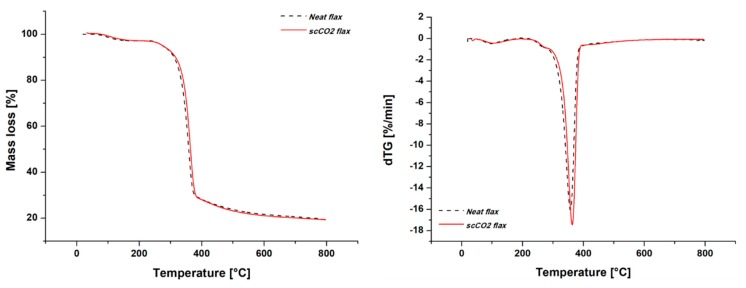
TGA thermograms and derivative thermogravimetry (dTG) curves of flax yarns before and after scCO_2_ treatment.

The influence of the supercritical CO_2_ treatment on the thermal properties of the flax yarns was investigated using thermogravimetric analysis. In Figure 4, the thermograms for the as-received and the treated flax yarns are reported.

The thermogram of the untreated flax yarns exhibited the typical three peaks in the derivative curve: the first mass loss, at about 60–120 °C, is related to the release of water occurring mostly in the amorphous region of cellulose; the second peak, at about 240–280 °C, is attributed to the decomposition of the non-cellulosic components such as pectin and hemicellulose; the third mass loss peak, at about 340–360 °C, is due to the cellulose degradation [45,46]. In the supercritical CO_2_ treated flax yarns, no significant changes in the thermal degradation behavior were detected, with the exception of a slight removal of hemicellulose and pectin components as supported by the higher values of the maximum degradation temperature and of the temperature of 10% weight loss compared to untreated flax yarns (Table 2).

Contrary to FT-IR results, the thermogravimetric analysis did not highlight a significant lignin degradation after supercritical CO_2_ treatment. In general, the weight loss of lignin occurs slowly over a broader temperature range than cellulose and hemicellulose components [47]. The lignin decomposition starts at relatively low temperatures, (200–275 °C), but the main process occurs around 400–450 °C, with the formation of by-products such as aromatic hydrocarbons, phenolics, and hydroxyphenolics [48]. Comparing the thermograms obtained for the untreated and treated flax yarns, no difference was highlighted in a temperature range around 400–450 °C. Even untreated flax fibers did not show significant degradation in the 400–450 °C temperature range. This can be ascribed to the low amount of lignin in flax fibers because it is usually dictated not only by the genetic pool of the plant but also by harvesting parameters. In fact, in order to make easier the extraction of flax fibers, it is preferable to keep the lignin content to a low level [1]. These results are in line with the ones obtained by François et al. on hemp fibers [38], where only a decrease in the third peak, related to partial lignin extraction, was observed in scCO_2_ treated samples. Indeed, hemp fibers are usually characterized by higher lignin content compared to flax fibers [1]. 

Mechanical characterization of untreated and scCO_2_ treated flax yarns was performed in order to investigate the effect of the treatment on their tensile behavior. The scatter of the tensile strength of flax yarns was statistically analyzed using a two-parameter Weibull distribution according to Equation (1):

(1) $$P_{r}\left(\sigma \right)=1-\exp\left[-{\left(\frac{\sigma }{\sigma_{0}}\right)}^{m}\right]$$

where *P_r_(σ)* is the cumulative probability of failure as a function of applied stress *σ*, *m* is the Weibull modulus (related to the dispersion of the data), and *σ*_0_ is the characteristic strength. Equation (2) provides the estimator, *P_f_*, used for the evaluation of failure probability:

(2) $$P_{f}=\frac{i-0.5}{N}$$

where *N* is the number of yarns tested and *i* is the rank of data point for each yarn.

The effects of the supercritical CO_2_ on the mechanical performance of flax yarns are reported in Table 3.

If the maximum force values obtained for untreated and treated flax yarns are compared, it is possible to highlight that the supercritical CO_2_ process does not produce a change of the load-bearing ability of the yarns. However, a slight reduction in the tensile strength value was observed, passing from a value of 236 ± 57 MPa to a value of 211 ± 36 MPa for the as-received and the CO_2_ treated flax yarns, respectively. This decrease is strictly dependent on the increase in the average diameter of yarns produced by the exposure to the supercritical fluid with higher individualization of fibers inside the yarn, as observed in single hemp fibers by Francois et al. [38]. Although not directly investigated in the present work, it is likely to suppose also a change in the twist angle of the yarns as a result of CO_2_ treatment. In addition, from results reported in Table 3, it is possible to notice that an increase in brittleness of flax yarns occurred after the exposure to the supercritical fluid. In the present case, the effects on the biochemical composition of the flax yarns are balanced by the slight increase in cellulose crystallinity, while the increased brittle behavior can be ascribed to the nucleation of small defects in the fiber cell wall after the depressurization step that prevented an extensive deformation [38].

**Figure 5:**
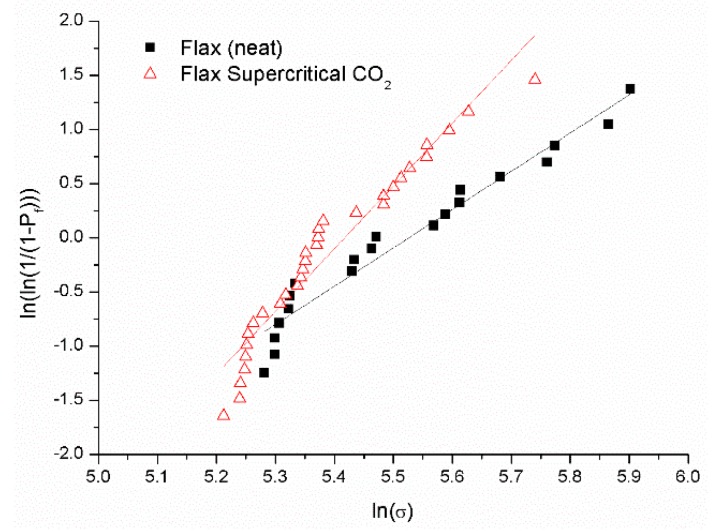
Weibull distribution plots for strength measured for untreated and supercritical CO_2_ treated flax yarns.

The experimental results, obtained for the as-received and the scCO_2_ treated flax yarns, and statistically analyzed using a two-parameter Weibull distribution, are reported in Figure 5. A quasi-linear trend was found for both untreated and treated flax yarns, which suggests that the yarn failure depends on the presence of a single population of flaws on the flax yarn surface. The supercritical CO_2_ treatment did not result in a change in the nature of the fiber defects.

### 2.2. Interfacial Quality Assessment by Single Yarn Fragmentation Test

**Figure 6:**
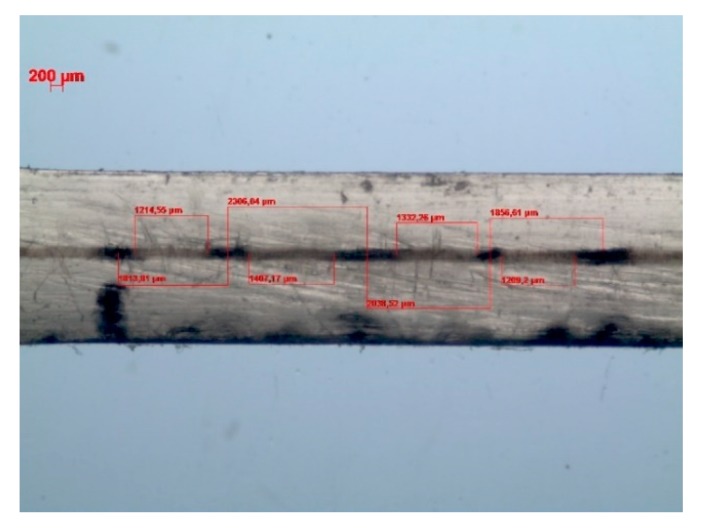
Typical optical micrograph of a fragmented flax yarn in an epoxy matrix: measurement of the yarn fragment length *l*.

Fragmentation tests have been performed for the untreated and the scCO_2_ treated flax yarns with both epoxy and vinylester resins. The critical fragment length was calculated according to Equation (3):

(3) $$l_{c}=\frac{4}{3}\cdot \bar{l}$$

where $\bar{l}$ is the average value of the fragment length. For each sample, the yarn diameter and fragment lengths were measured in the gauge length zone, as depicted in Figure 6. In particular, the fragment lengths were determined as the average values between the internal and the central points of the breaking zones. In a previous publication [49], the authors demonstrated by micro-CT that the dark region around each yarn breakpoint represents the debonding length (*l_debonding_*) at the flax yarn/matrix interface.

**Figure 7:**
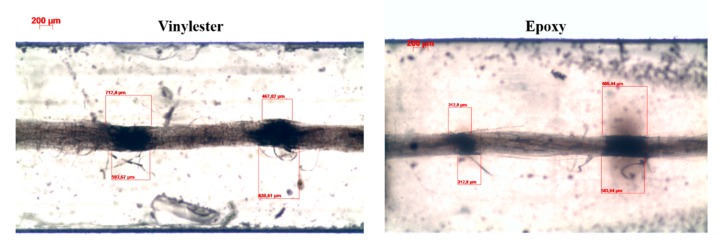
Typical optical micrographs of fragmented flax yarns in vinylester and epoxy matrices: measurement of the debonding length (*l_debonding_*).

These length values were measured by optical microscopy (Figure 7) and the results are summarized in Table 4. For each family of single yarn composites, at least 10 fragmentation tests were carried out. From the results, it is possible to observe that the scCO_2_ treatment produced a slight reduction in the critical length of flax yarns in both epoxy and vinylester matrices. However, an assessment of the interfacial adhesion quality entirely based on the comparison of critical length is not sound in the present case, as the flax yarn strength changed after the surface treatment [50]. There is a strong relationship between the fragment length value and the yarn tensile strength: the greater the failure strength of yarn, the greater the length needed to reach this load and provide fragmentation. For this reason, it should not be proper to evaluate the differences in interfacial bond of the different flax yarn/epoxy and flax yarn/vinylester systems in terms of critical fragment length. However, a comparison between the different values found for the same flax yarn family with the epoxy and vinylester matrices is feasible. The two thermoset systems exhibited different results. From values reported in Table 4, it is possible to infer how the flax/epoxy samples are characterized by smaller values of critical lengths than the flax/vinylester ones, indicating a better adhesion quality. This difference between the epoxy and vinylester systems can be related to the different abilities of the two polymer resins to wet the surface of flax yarns.

In fact, it is important to highlight that the vinylester resin selected for this experimental work is a quite innovative styrene-free resin in which the styrene monomer is replaced by 1,4-butanediol dimethacrylate (BDDMA). Recently, Bénéthuilière et al. [51] investigated the potential of a styrene-free resin on the adhesion level of E-glass fibers by performing the microbond test. As in the present study, the BDDMA was used as a reactive diluent for the styrene-free resin and the results were compared with those found with a conventional vinylester resin. The authors reported that the styrene-free resin displayed a lower ability than a conventional vinylester resin to wet E-glass fibers having a vinylester-compatible sizing. This phenomenon is due to the fully dispersive nature of the styrene-free resin, while the E-glass fibers have a much higher polar characteristic. Flax yarns are characterized by a strongly polar behavior that can explain the low compatibility with the fully dispersive styrene-free vinylester resin.

The interfacial shear strength value (IFSS) was estimated in order to describe the yarn/matrix interface adhesion quality in accordance with the model proposed by Kelly and Tyson [52]:

(4) $$IFSS=\frac{\sigma_{f}\left(l_{c}\right)\cdot d}{2\cdot l_{c}}$$

where *d* is the yarn diameter, *l_c_* is the critical fragment length, and *σ_f_*_(*lc*)_ is the yarn strength at a length equal to the critical filament length. This strength value was determined by extrapolation of the Weibull cumulative distribution function obtained for only one given yarn gauge length *l*_0_ (Equation (5)).

(5) $$\sigma_{f}\left(l_{c}\right)=\;\sigma_{f}\left(l_{0}\right){\left(\frac{l_{0}}{l_{c}}\right)}^{-\frac{1}{m}}$$

where *m* is the shape parameter of the Weibull distribution for the tested gauge length *l*_0_ of 40 mm, *l_c_* is the critical fragment length, and *σ*_*f*(*l*0)_ is the strength value at the gauge length. The results of this analysis are summarized in Table 5.

The difficulty in adapting the fragmentation test to yarns stems from the cross-sectional area measurement of the yarn that typically includes voids between the fibers that are then replaced by matrix during the impregnation step of the single yarn. Therefore, IFSS values were calculated but in the present case, their numerical values need to be considered only on a qualitative level to rank the different flax yarns.

IFSS values for neat flax yarns confirm the better interfacial adhesion with the epoxy matrix. Particularly interesting are the interfacial shear strength values obtained for the scCO_2_ treated flax yarn/vinylester system. This treatment did not significantly reduce the tensile properties of flax yarns (Table 3). It is, therefore, possible to directly compare the IFSS values obtained after the scCO_2_ treatment with those found for the as-received flax yarn. Considering the flax/vinylester system, a slight increase in the interfacial adhesion is produced after the exposure of flax yarn to the supercritical fluid. On the contrary, a decrease in IFSS value is reported with the epoxy matrix. However, as previously mentioned, the exposure to the supercritical fluid caused an increase in the average diameter of yarns, which resulted in a slight reduction in the tensile strength value. This reduction explains the decrease in the IFSS values found for the flax/epoxy system, being the IFSS results strictly dependent on any variation in the mechanical properties of flax yarns produced by the surface modification treatment. The values reported in Table 5 highlight that the two thermoset systems exhibited different IFSS results. The flax/epoxy samples are characterized by higher values of interfacial shear strength than the flax/vinylester ones. This result confirmed once again the better adhesion quality between flax yarn and epoxy resin than that observed for the vinylester matrix.

The issues previously raised about the use of the fragmentation test at the yarn scale can be addressed by considering another parameter, i.e., the length of the debonding zone between the flax yarns and the matrix [53,54]. The higher is the debonding length, the lower is the adhesion quality between the filament and the polymer matrix (Figure 7), being this value independent of the constraints that should be met for a valid SFFT (for instance, no change in mechanical properties of the fiber after surface treatment). The results (Table 4) showed that the scCO_2_ treatments produced a decrease in the length of the debonding zone, promoting the interfacial adhesion of flax yarns with the epoxy and, in particular, with the vinylester resin. This result is consistent with the critical filament length values previously reported (Table 4) and it points out that the supercritical carbon dioxide fluid can successfully promote the adhesion of flax yarns with the vinylester matrix. This positive effect produced on the flax yarn/matrix adhesion can be linked to the non-cellulosic constituents’ removal, such as lignin, pectin, and hemicellulose, occurred during the scCO_2_ process and confirmed by TGA and FT-IR measurements. The debonding length values found for the epoxy matrix samples are lower than the values found for the vinylester matrix ones. These results are again consistent with the critical filament length and IFSS values and highlight the better compatibility of flax yarns with the epoxy matrix than with the vinylester one.

**Figure 8:**
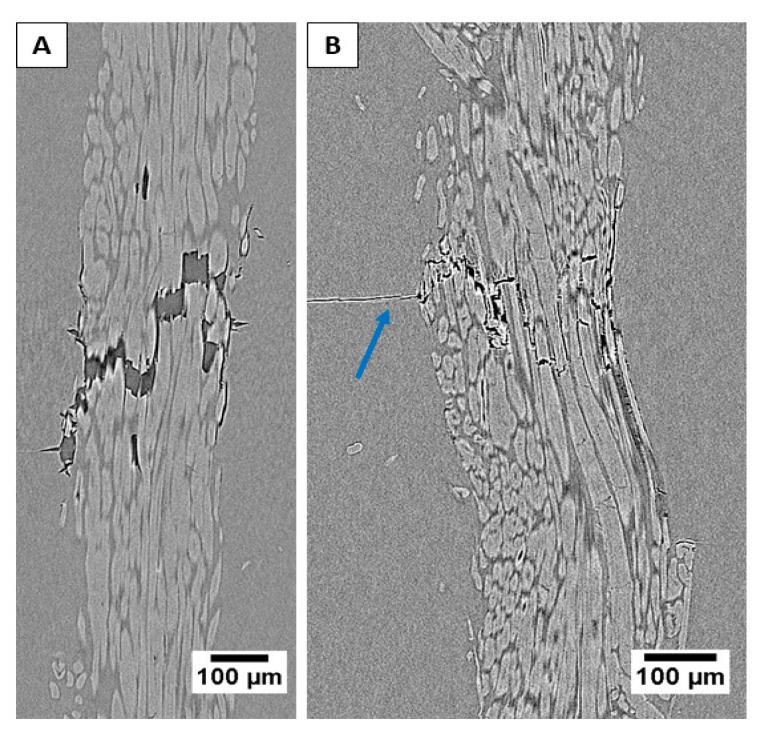
Debonding length observation by micro-computed tomography. High-resolution micro-CT images for supercritical CO_2_ treated flax/epoxy (**A**) and flax/vinylester (**B**) single yarn samples.

The debonding length results were also confirmed by micro-computed tomography, with which a post-mortem examination of the single yarn composites enabled precise measurement of the yarn/matrix debonding zone. The debonding length was identified by the black voxels corresponding to voids between the periphery of the flax yarn and the matrix (Figure 8). If compared to untreated flax yarns [49], the microtomographic images for the supercritical CO_2_ treated flax/matrix samples confirmed the decrease in debonding length with both epoxy and vinylester resin. The presence of some resin cracks inside the vinylester matrix, identified by a blue arrow in Figure 8B, supports the increase in compatibility and the enhancement in load transfer ability between the treated flax yarn and the vinylester polymer matrix.

## 3. Materials and Methods

### 3.1. Raw Materials

As regards the plant reinforcement, a Biotex flax fabric (*Linum usitatissimum*), supplied by Composites Evolution (Chesterfield, UK), was used. This fabric, a 2 × 2 twill fabric (200 g/m^2^), is commercialized without any specific sizing and individual flax yarns were carefully extracted by hand. Two different thermoset matrices were included in this study, an epoxy and a vinylester resin. As regards the epoxy system, an Epoxy Prime 27 infusion resin and PRIME 20 slow hardener by GURIT (Newport, UK) were used. The curing process was carried out with a recommended mixing ratio of 100:28 by weight. In order to reduce the risks derived from styrene, a quite innovative styrene-free vinylester resin was selected. Specifically, the Advalite VH-1207 vinylester infusion resin, supplied by Reichhold (London, UK), was chosen. The Norpol Peroxide PMEC N24 hardener, supplied by Reichhold, was used as curing agent and the curing process was carried out with a mixing ratio of 1 phr. The resin is pre-accelerated for room temperature cure.

### 3.2. Supercritical Carbon Dioxide (scCO_2_) Treatment of Flax Yarns

**Figure 9:**
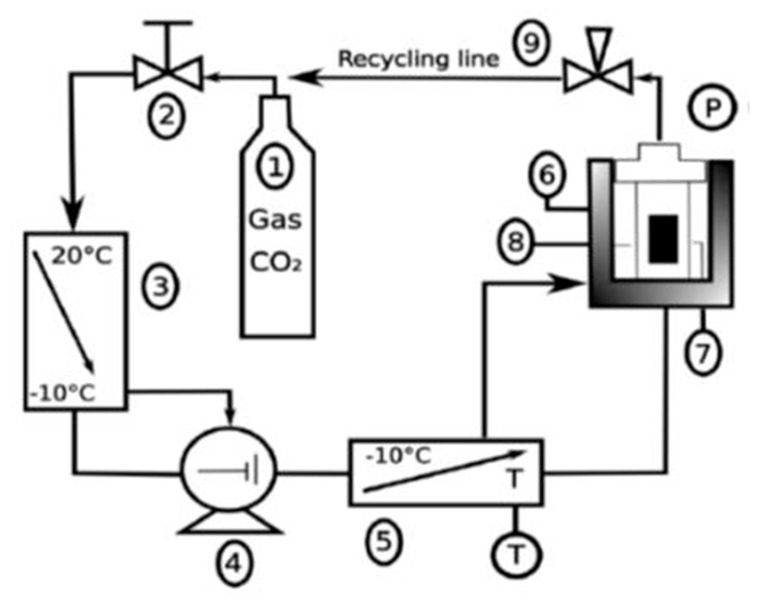
Schematic diagram of the supercritical equipment provided by Separex.

Flax yarns were treated using a piece of equipment supplied by Separex (Nancy, France). The flax yarns were first placed in a temperature and pressure-controlled enclosure. A schematic representation of the equipment setup used is reported in Figure 9. Gaseous carbon dioxide (1) was extracted from a bottle by a valve (2) at the beginning of the process. It was then cooled to a liquid state (3) and directed toward a pump (4), which controlled the pressure. When it reached the pressure needed for the supercritical state (up to the critical point of 73.8 bar), the carbon dioxide was heated by a heat exchanger (5). At the supercritical state, the CO_2_ was directed toward the autoclave (6), which contained the flax yarn samples (7). Thermocouples were used to control the temperature (8). The treatment of flax in autoclave was carried out under high temperature and pressure conditions (130 °C, 50 bar) for a duration of 170 min. When the treatment was finished, the carbon dioxide was recovered (9), cooled, and directed toward a recycling line. The amount of CO_2_ injected in the reactor was measured and controlled using a balance. After the process, the reactor was depressurized from 150 bar to 0 bar to allow its opening and the samples were stored at room temperature for cooling.

### 3.3. Tensile Testing of Flax Yarns

Tensile properties of untreated and scCO_2_ treated flax yarns were determined by single yarn tensile tests in accordance with ASTM C-1557 standard test method [21,49]. Tensile tests were carried out at room temperature using a Zwick/Roell Z010 machine (Ulm, Germany) equipped with a 1 kN load cell. Each test was performed in displacement control at a cross-head speed of 2 mm/min. Individual flax yarns were carefully separated by hand from the fabric and glued (Loctite™ Gel Superglue, Milan, Italy) onto a card tab with a central window cut out to match the gauge length of 40 mm. At least 20–30 yarns were tested for each type of yarns. Before testing, all yarns were conditioned at 45 °C for 24 h. Before each test, the yarn diameter was evaluated as the average of at least ten measurements along the gauge length by optical microscopy.

### 3.4. Fourier Transform Infrared Spectroscopy Analysis (FT-IR)

The chemical composition of the as-received and scCO_2_ treated flax yarns has been studied by Fourier-transform infrared (FT-IR) analysis. Infrared measurements were carried out with a Bruker Vertex 70 spectrometer (Bruker Optik GmbH, Ettlingen, Germany) equipped with a single reflection Diamond ATR cell. Spectra were recorded with a 3 cm^−1^ spectral resolution in the mid-infrared range (400–4000 cm^−1^) using 512 scans.

### 3.5. Thermogravimetric Analysis (TGA)

The thermal stability of untreated and treated flax yarns has been investigated using a SetSys Evolution (Setaram Instrumentation, Caluire France) thermogravimetric analyzer. The different samples were placed in an alumina pan and heated at a rate of 10 °C/min to a maximum temperature of 800 °C in nitrogen atmosphere. Weight changes versus temperature and the derivative of weight changes versus temperature have been recorded.

### 3.6. Single Yarn Fragmentation Test

In order to assess the interfacial properties of untreated and scCO_2_ treated flax yarns with epoxy and vinylester matrices, the well-known single fiber fragmentation test (SFFT) was slightly modified and adapted to test single yarns, as reported in our previous publications [21,49]. A dedicated metallic mould has been used for manufacturing composite specimens reinforced with a single flax yarn aligned along the applied tensile load. Each flax yarn was positioned in the mould and held in place by adhesive tape. Before matrix casting, flax yarns were conditioned at 45 °C for 24 h. The specimens showed a dog bone shape with reduced gage section characterized by a length of 15 mm, a thickness of 2 mm, and a width of 3 mm. An Instron E1000 ElectroPuls test machine (Norwood, MA, USA) with a load cell of 2 kN was used for the fragmentation test with a crosshead speed of 0.005 mm/min. Before each test, the yarn diameter was evaluated as the average of at least ten measurements along the gauge length by optical microscopy (Zeiss, Oberkochen, Germany. The test was carried out at a rather low loading rate and the loading phase was stopped if the specimen failed, or when the fragmentation saturation level was achieved. This last case was defined when no new filament breaks appeared during a subsequent strain increase by 0.5%, as reported in literature [55].

In order to be sure to reach the saturation level, epoxy- and vinylester-based specimens were loaded up to strains higher than 9% and 11%, respectively. All flax yarns were characterized by a strain at failure at least three and two times smaller than the failure strain of both epoxy and vinylester resins, respectively, thus ensuring the compliance with the Kelly–Tyson model requirements [52].

### 3.7. X-ray Microtomography

An UltraTom CT scanner manufactured by RX Solutions (Chavanod, France) has been used for the analysis of single yarn composites with a resolution of 1.5 μm, an accelerating voltage of 50 kV, and a beam current of 157 µA.

### 3.8. Optical and FE-SEM Observations

The flax yarn diameters, as well as the fragment lengths, were measured by using a ZEISS Axio Imager optical microscope (Oberkochen, Germany). A morphological investigation of scCO_2_ treated and untreated flax yarns was carried out using a field-emission gun scanning electron microscope (FE-SEM) Zeiss Auriga. All specimens were sputter-coated with chromium prior to FE-SEM observation.

## 4. Conclusions

Commercially available flax yarns were subjected to a supercritical CO_2_ treatment with the aim of modifying their interfacial adhesion with two different thermoset matrices, namely an epoxy and a vinylester resin. This green treatment was found to alter the biochemical composition of flax fibers, as confirmed by FT-IR and TGA analyses, which highlighted the preferential removal of hemicellulose, lignin, and pectin with a general cleaning effect of fiber surface but without degradation of their thermal stability. These constituents are mostly responsible for the highly polar behavior of flax fibers, thus suggesting a potential reduction of moisture sensitivity and a general increase in fiber/matrix compatibility. This assumption was confirmed by single yarn fragmentation tests, where the debonding and critical fragment length values were reduced after the exposure to the supercritical fluid, in particular with reference to the fully dispersive vinylester matrix. These preliminary results suggest the potential of this treatment as an attractive green method to tailor the surface compatibility of lignocellulosic fibers, even though there is still room for optimizing the process parameters (temperature, pressure, and exposure time) and their effects on fibers’ physico-mechanical properties.

## Figures and Tables

**Table 1:** Assignment of the main ATR-FTIR bands of flax fibers [21].

Wavenumber (cm^−1^)	Band Assignments *
3700–3000	*ν*OH
2990–2754	*ν*CH of cellulose and hemicellulose
1742	*ν*C=O of hemicellulose
1637	*δ*HOH of water in crystalline cellulose
1597, 1514, 1450	*ν*C=C and *d*CH in methyl, methylene and methoxyl groups of lignin
1427, 1368, 1314	*δ*CH_2_, *δ*CH of cellulose and *ω*CH_2_ in cellulose and hemicellulose
1278	*τ*C-H_2_ of cellulose
1248, 1160, 1104, 1029	*ν*C-O-C and *ν*C-C of polysaccharide components (mainly cellulose)
1053	*ν*C-OH of cellulose and hemicellulose
897	β-glycosidic linkages between the sugars units in cellulose

* *ν* = stretching; *δ* = bending; *d* = deformation; *ω* = wagging; *τ* = twisting.

**Table 2:** Results of the thermogravimetric analysis for the untreated and scCO_2_ treated flax yarns.

	Maximum Degradation Temperature [°C]	Temperature of 10% Weight Loss [°C]
Flax yarn (neat)	357.2	311.3
Flax supercritical CO_2_	363.4	313.9

**Table 3:** Tensile properties of untreated and scCO_2_ treated flax yarns and the corresponding Weibull parameters.

Specimen	F_max_ [N]	Diameter [µm]	σ_f_ [MPa]	ε_f_ [%]	Characteristic Strength σ_0_ [MPa]	Weibull Modulus, m
Neat flax	19.8 ± 4.8	327 ± 96	236 ± 57	3.40 ± 0.42	257	5.02
scCO_2_ flax	20.4 ± 3.5	350 ± 68	211 ± 36	1.40 ± 0.15	226.1	7.10

**Table 4:** Critical fragment length and debonding length values for the different single yarn composites.

Matrix	Flax Yarn	l_c_ [µm]	l_debonding_ [µm]
Epoxy	Neat	2687 ± 631	444 ± 49
	scCO_2_	2587 ± 623	429 ± 39
Vinylester	Neat	3942 ± 807	830 ± 343
	scCO_2_	3345 ± 490	673 ± 145

**Table 5:** Strength values at critical fragment length and interfacial shear strength values (IFSS) for the different flax/epoxy and flax/vinylester systems.

Matrix	Flax Yarn	σ_f_ (l_c_) [MPa]	IFSS [MPa]
Epoxy	Neat	406 ± 18	19.3 ± 3.7
	scCO_2_	312 ± 10	15.7 ± 2.1
Vinylester	Neat	376 ± 15	13.9 ± 2.8
	scCO_2_	300 ± 6	14.3 ± 2.6
